# Supplementary material for: Addressing Cognitive Bias in Adolescents with Neurodevelopmental Disorders Using 3-D Animated Serious Games
Source: Pediatr Rep. 2025 Feb 25;17(2):28. doi: 10.3390/pediatric17020028 (PMC11932302; doi:10.3390/pediatric17020028)
Supplement: Supplementary file 1 [file pediatrrep-17-00028-s001.zip › pediatrrep-3403800-supplementary S2.pdf]

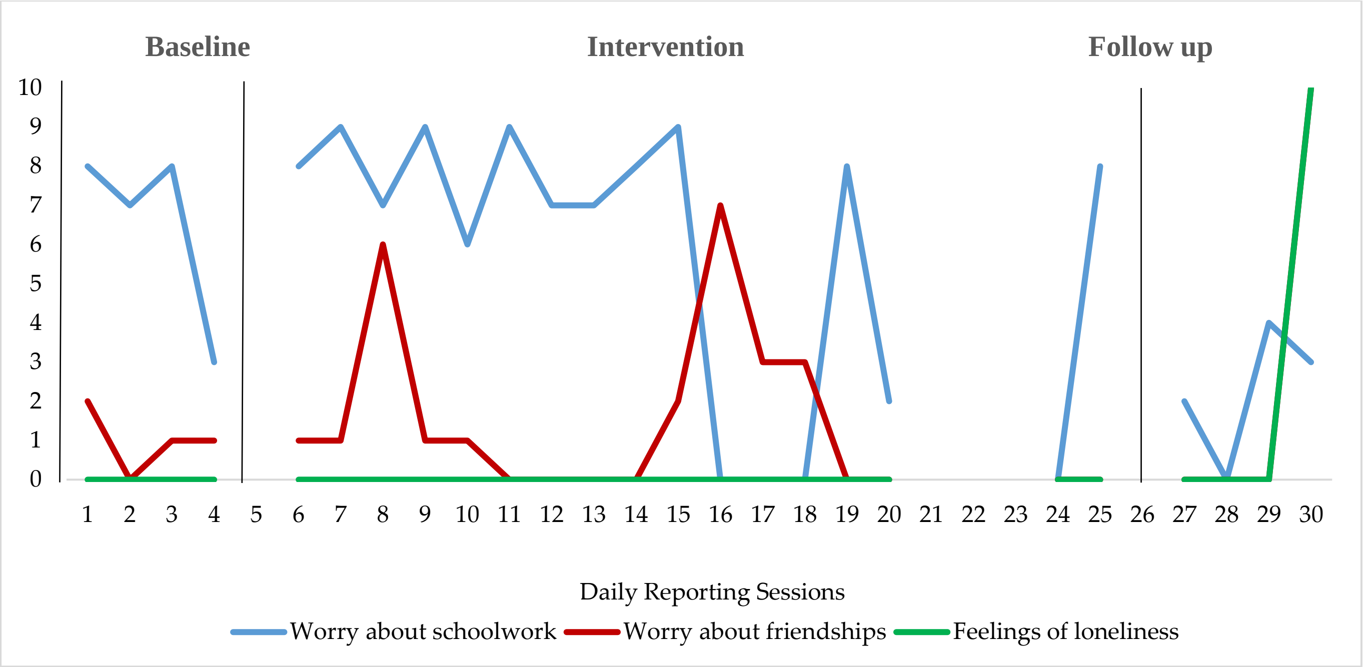

Participant 1

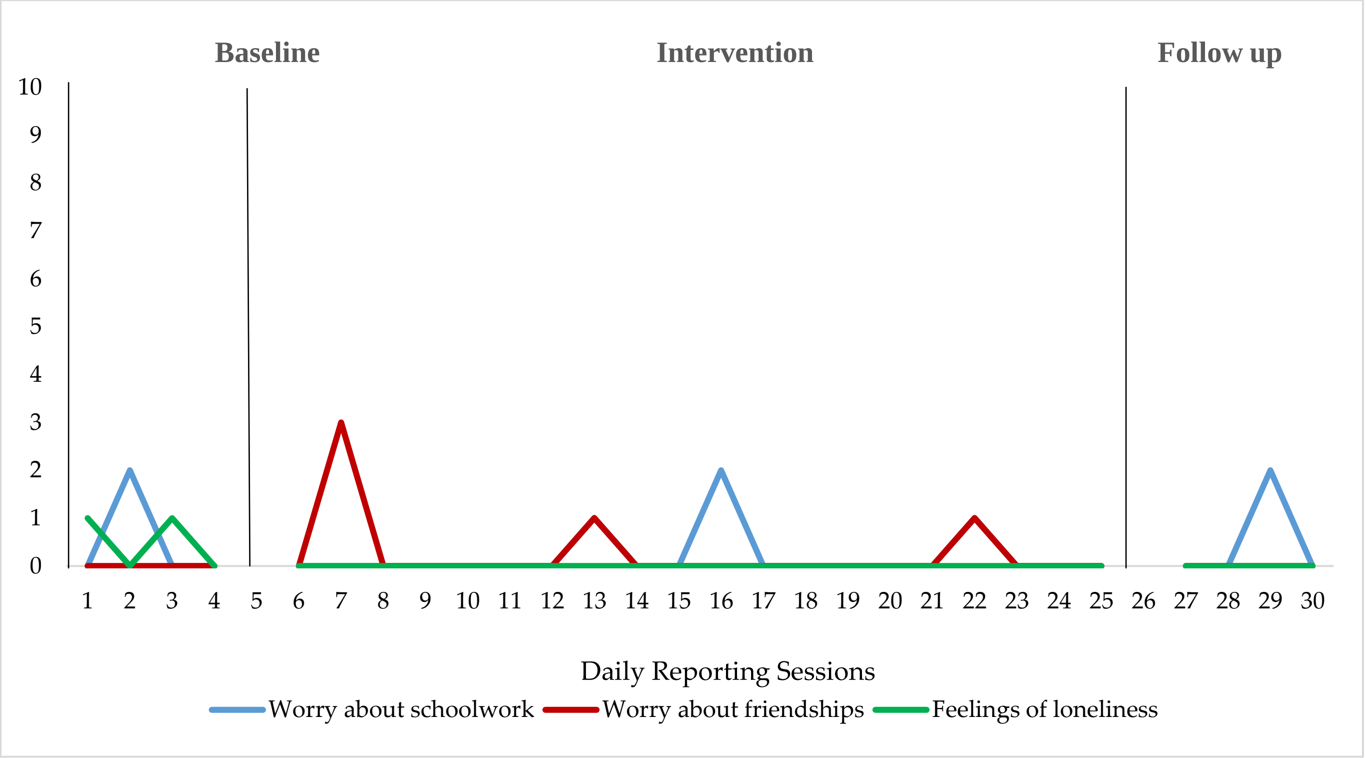

Participant 2

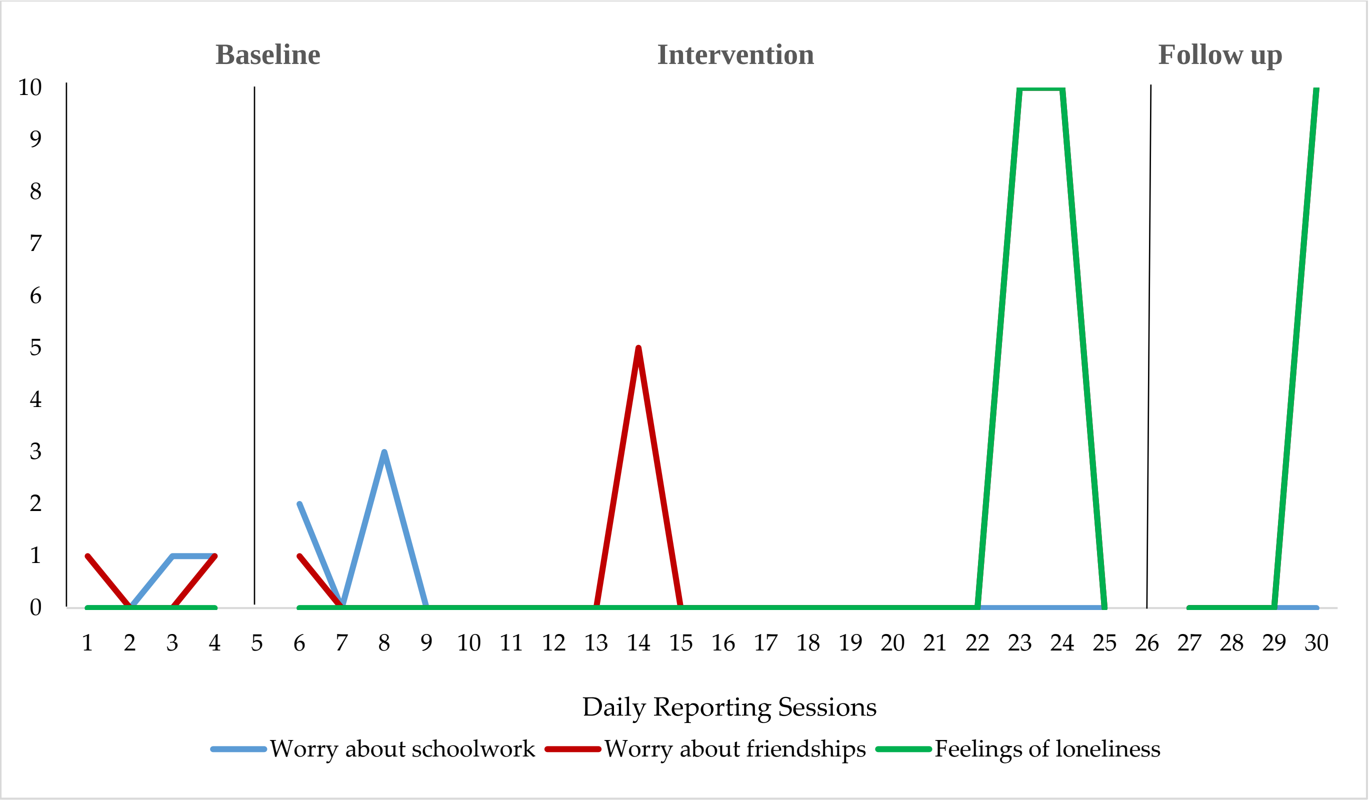

Participant 3

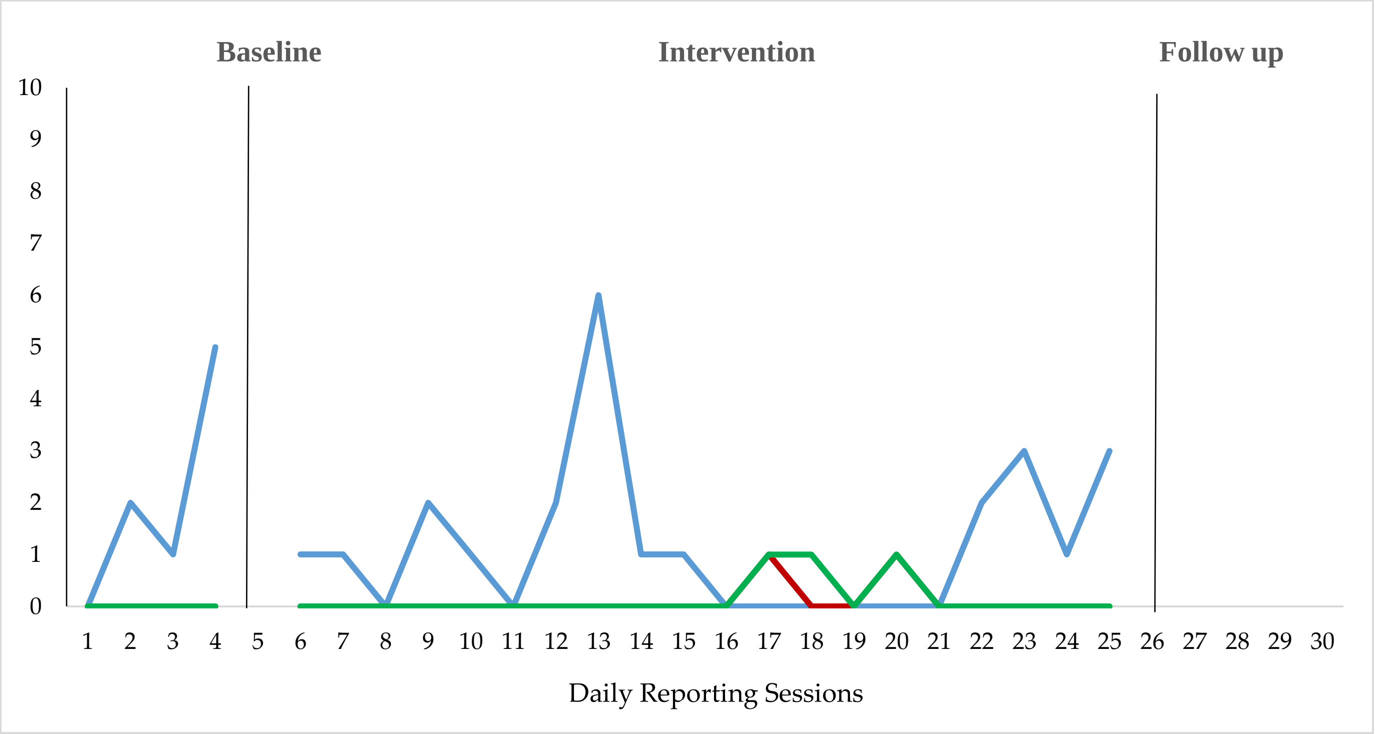

Participant 4

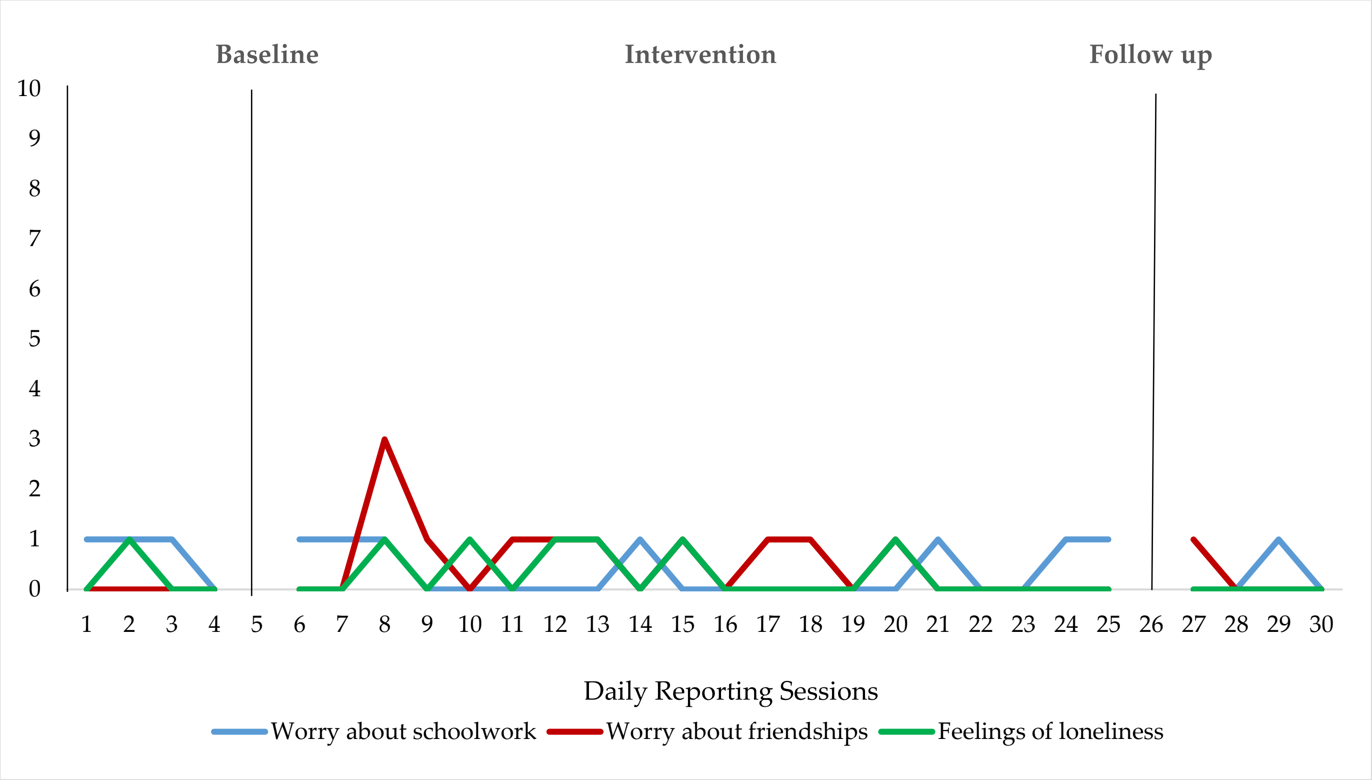

Participant 5

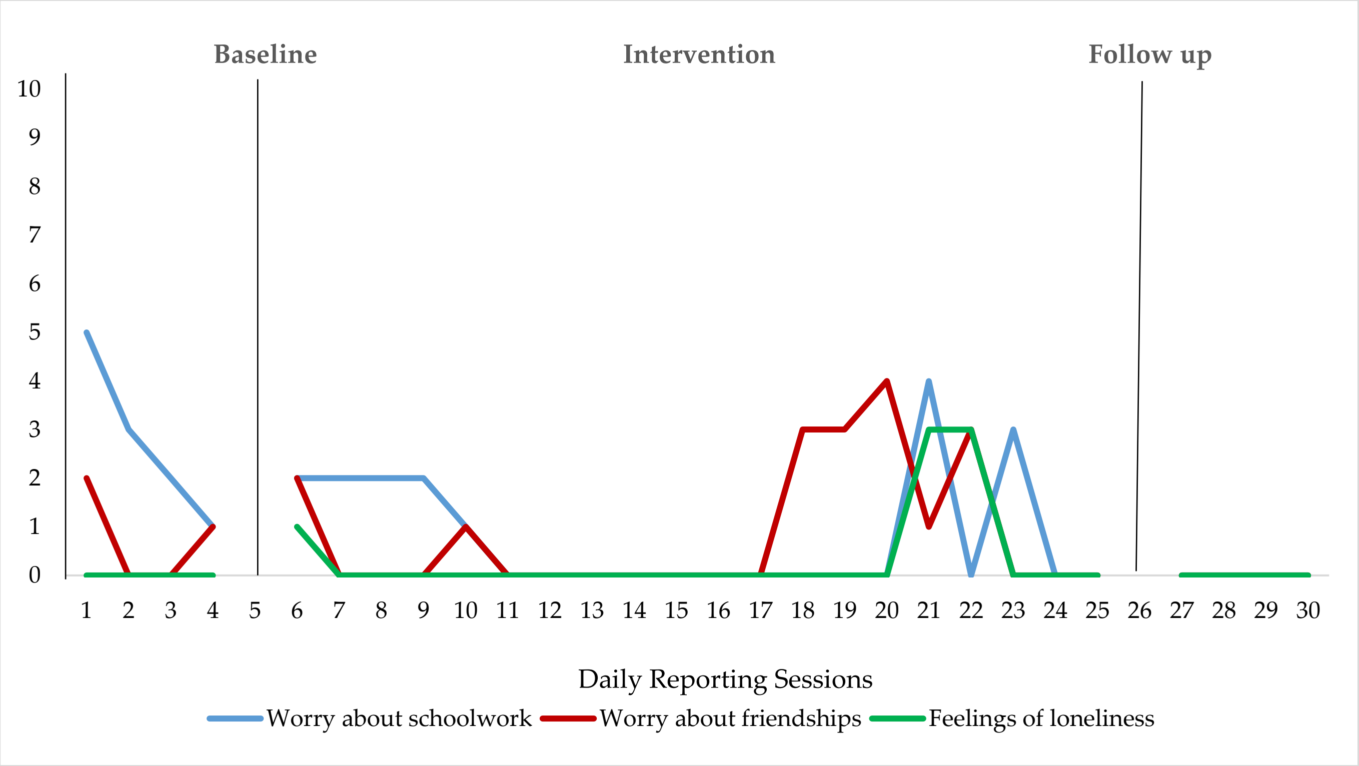

Participant 6

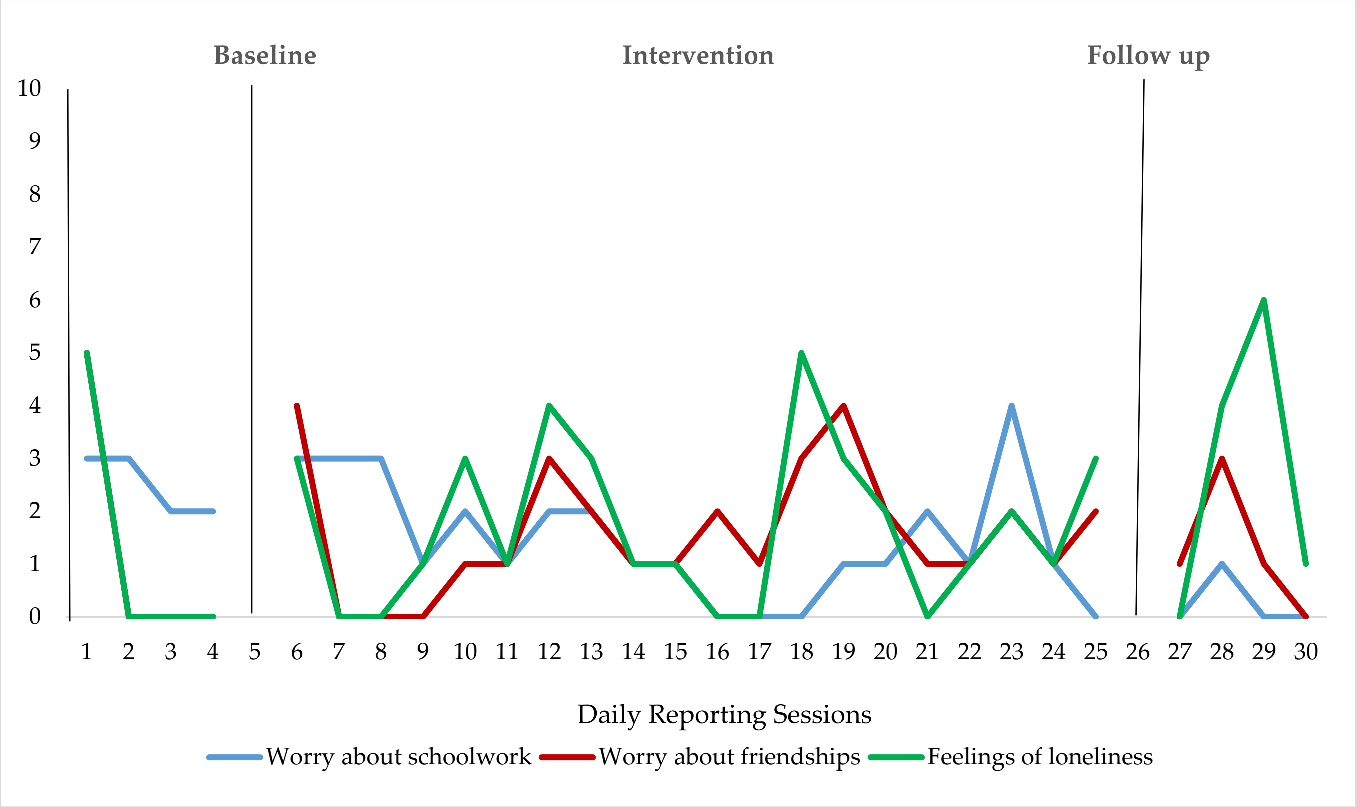

Participant 7

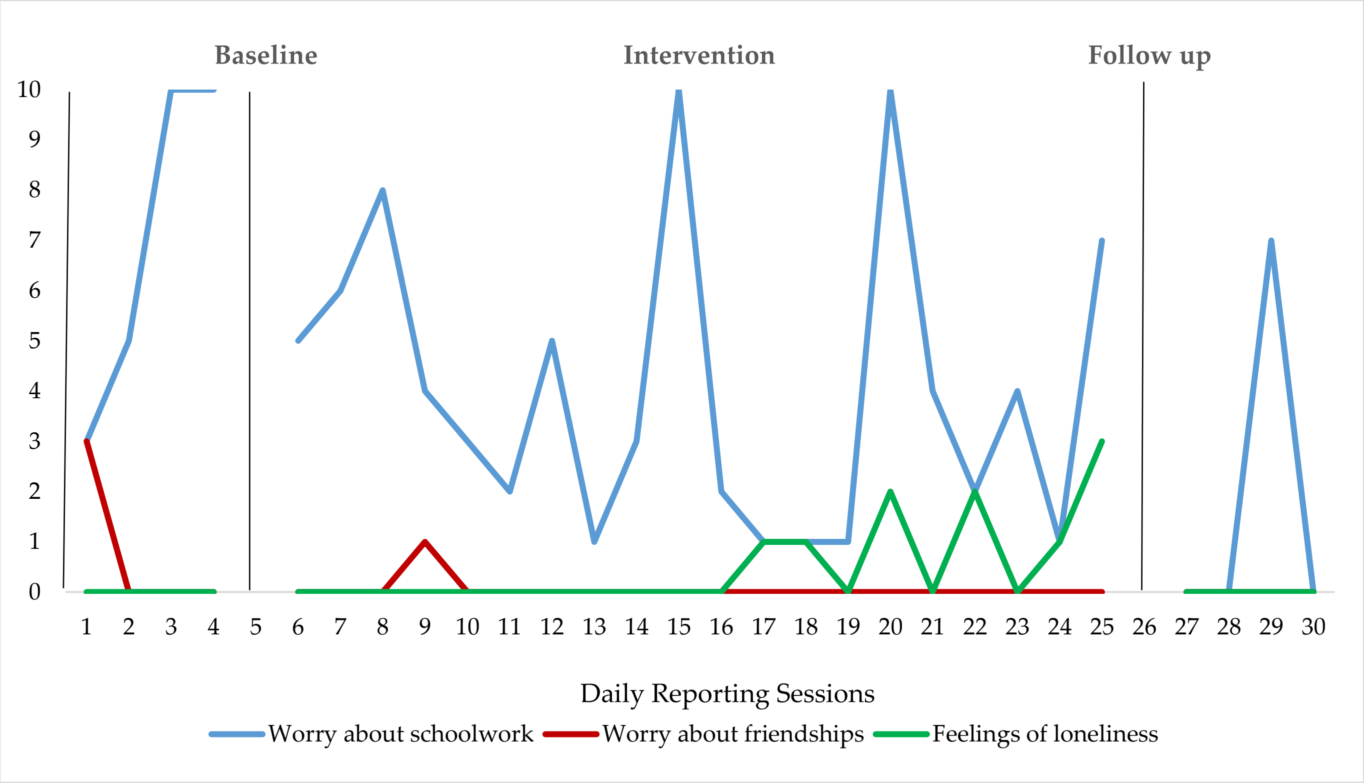

Participant 8

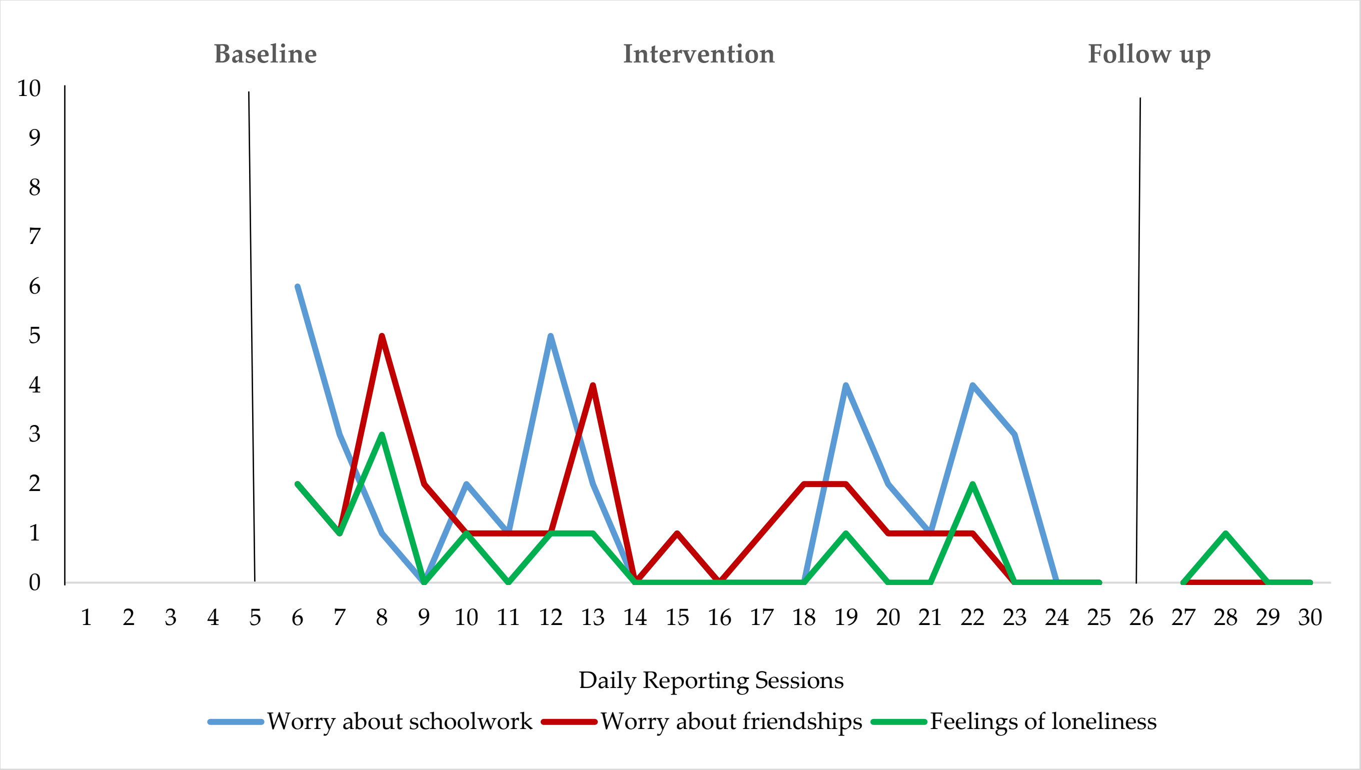

Constant Series Control

Supplementary Figure S2. Multiple baseline visual trends for worry about schoolwork, worry about friendships, and feelings of loneliness.
