# Supplementary figures and images for: Addressing Cognitive Bias in Adolescents with Neurodevelopmental Disorders Using 3-D Animated Serious Games
Source: Pediatr Rep. 2025 Feb 25;17(2):28. doi: 10.3390/pediatric17020028 (PMC11932302; doi:10.3390/pediatric17020028)

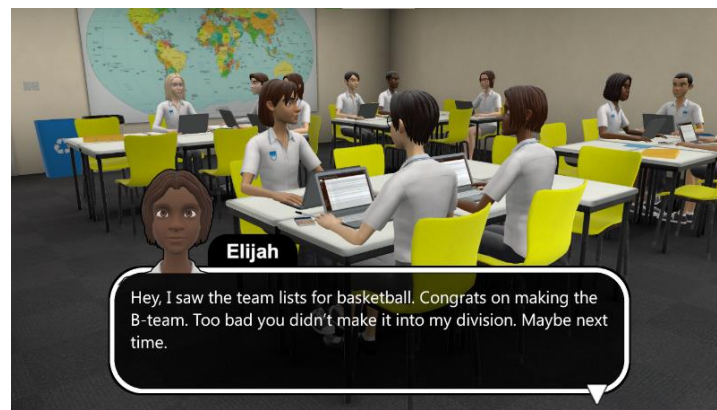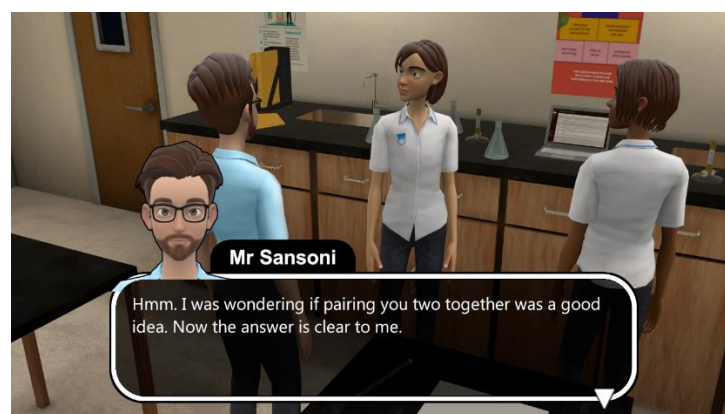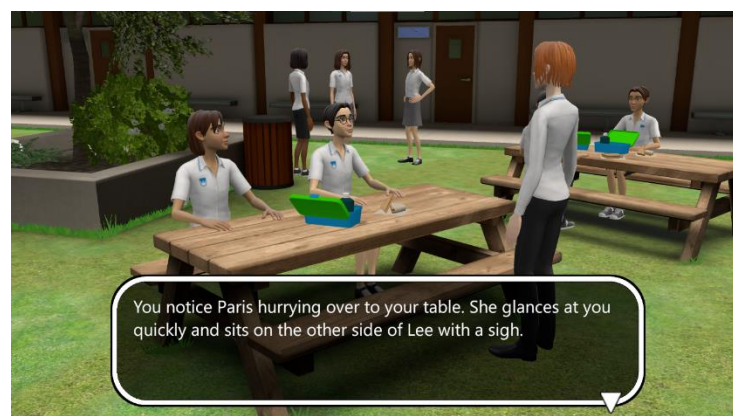

**Figure S1.** Example screenshots from Minds Online.

Supplement: Supplementary file 1 [file pediatrrep-17-00028-s001.zip › pediatrrep-3403800-supplementary S1.pdf]
